# Crystal structure, Hirshfeld surface analysis, and calculations of inter­molecular inter­action energies and energy frameworks of 1-[(1-hexyl-1*H*-1,2,3-triazol-4-yl)meth­yl]-3-(1-methyl­ethen­yl)-benzimidazol-2-one

**DOI:** 10.1107/S2056989024008703

**Published:** 2024-09-30

**Authors:** Zakaria El Atrassi, Zakaria Benzekri, Olivier Blacque, Tuncer Hökelek, Ahmed Mazzah, Hassan Cherkaoui, Nada Kheira Sebbar

**Affiliations:** a Laboratory of Heterocyclic Organic Chemistry Medicines Science Research Center Pharmacochemistry Competence Center Mohammed V University in Rabat Faculté des Sciences Av Ibn Battouta BP 1014 Rabat Morocco; b University of Zurich, Department of Chemistry B, Winterthurerstrasse 190, 8057 Zurich, Switzerland; c Department of Physics, Hacettepe University, 06800 Beytepe, Ankara, Türkiye; d Science and Technology of Lille USR 3290, Villeneuve d’ascq cedex, France; e Laboratory of Organic and Physical Chemistry, Applied Bioorganic Chemistry Team, Faculty of Sciences, Ibnou Zohr University, Agadir, Morocco; f Laboratory of Plant Chemistry Organic and Bioorganic Synthesis Faculty of Sciences Mohammed V University in Rabat 4 Avenue Ibn Battouta BP 1014 RP Rabat Morocco; Vienna University of Technology, Austria

**Keywords:** crystal structure, benzimidazol-2-one, triazole, C—H⋯π(ring) inter­action, hydrogen bond

## Abstract

The benzimidazole moiety in the title mol­ecule, C_19_H_25_N_5_O, is almost planar and oriented nearly perpendicular to the triazole ring. In the crystal, C—H⋯O hydrogen bonds link the mol­ecules into a network structure. There are no π–π inter­actions present but two weak C—H⋯π(ring) inter­actions are observed. A Hirshfeld surface analysis of the crystal structure indicates that the most important contributions for the crystal packing are from H⋯H (62.0%), H⋯C/C⋯H (16.1%), H⋯N/N⋯H (13.7%) and H⋯O/O⋯H (7.5%) inter­actions. Evaluation of the electrostatic, dispersion and total energy frameworks indicate that the stabilization is dominated *via* the dispersion energy contributions in the title compound.

## Abstract 2

In the title mol­ecule, the benzimidazole moiety is oriented almost perpendicular to the triazole ring. In the crystal, C—H⋯O hydrogen bonds link the mol­ecules into a network structure.

## 1. Chemical context

Research into the properties of heterocyclic compounds, in particular benzimidazolo­nes, has become increasingly important. These compounds possess unique structural features and have shown a wide range of biological activities, including anti­proliferative (Guillon *et al.*, 2022 ▸), anti­bacterial (Al-Ghulikah *et al.*, 2023 ▸; Saber *et al.*, 2020 ▸; Ibrahim *et al.*, 2021 ▸), anti­cancer (Dimov *et al.*, 2021 ▸), anti­viral (Ferro *et al.*, 2017 ▸) and anti­depressant (Clayton *et al.*, 2020 ▸) properties, and activities related to Alzheimer’s disease (Mo *et al.*, 2020 ▸). Our research group recently made significant advances in synthesizing compounds that combine the 1,2,3-triazole moiety with benzimidazol-2-one derivatives.

**Figure 1:**
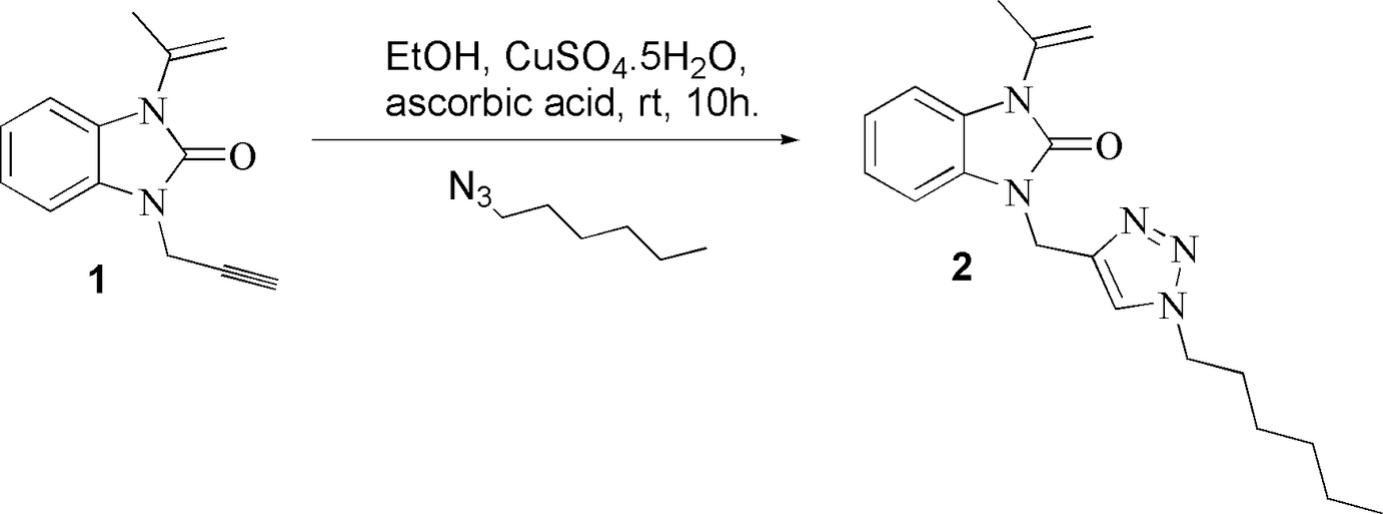
Reaction scheme for the synthesis of benzimidazole derivatives using the CuAAC method.

Here we provide details of the synthesis and the mol­ecular and crystal structures of 1-[(1-hexyl-1*H*-1,2,3-triazol-4-yl)meth­yl]-3-(1-methyl­ethen­yl)benzimidazol-2-one, C_19_H_25_N_5_O. We have synthesized this compound using click chemistry, in particular by applying copper-catalysed azide–alkyne cyclo­addition (CuAAC). This approach not only ensures efficiency in the synthesis process but also facilitates the formation of complex mol­ecular structures (Fig. 1 ▸). We also carried out Hirshfeld surface analysis and calculations of the inter­molecular inter­action energies and energy frameworks.
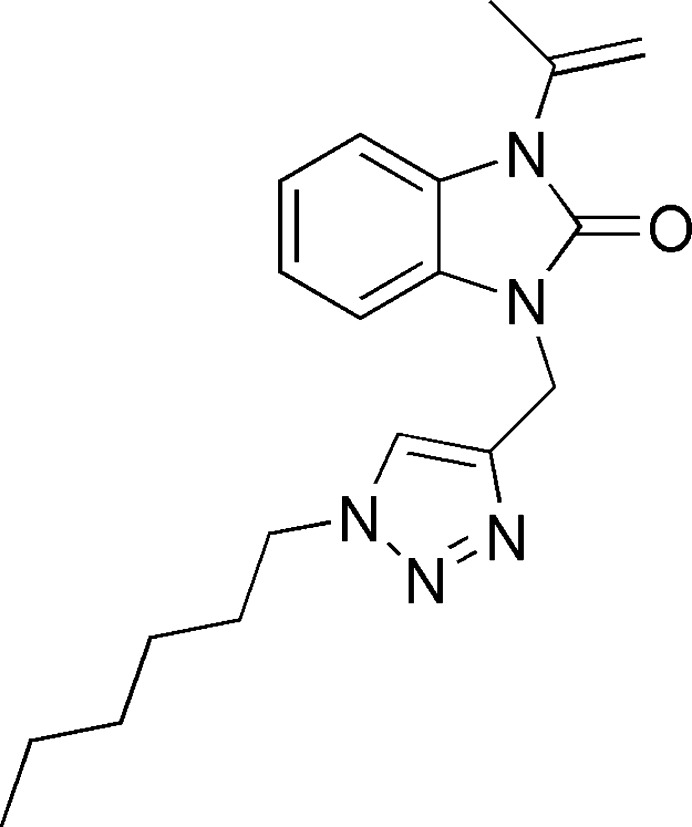


## 2. Structural commentary

**Figure 2:**
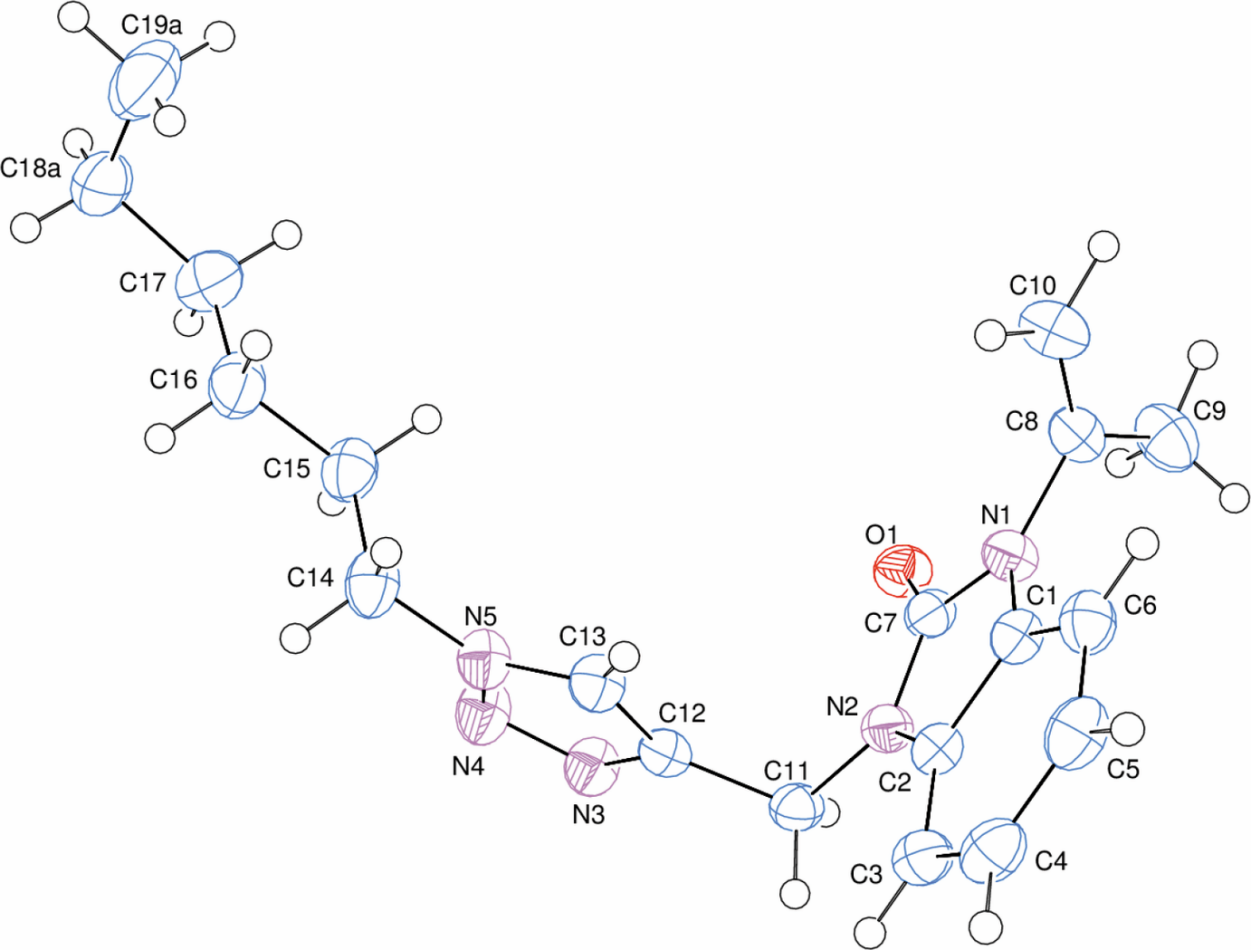
The mol­ecular structure of the title compound with displacement ellipsoids drawn at the 50% probability level. For clarity, only the major occupancy component of the disordered terminal propyl moiety of the hexyl chain is shown.

The mol­ecular structure of the title compound is shown in Fig. 2 ▸. The benzimidazole moiety is almost planar, the planar *A* (C1–C6) and *B* (N1/N2/C1/C2/C7) rings being oriented at a dihedral angle of 0.86 (5)°. Atoms O1 and C8 are 0.370 (10) Å and −0.0404 (16) Å, respectively, away from the least-squares plane of ring *B*. The planar triazole ring, *C* (N3–N5/C12/C13), is oriented almost perpendicular with respect to the benz­imidazole moiety at a dihedral angle of 87.57 (4)°, with atoms C11 and C14 lying 0.0044 (14) and 0.0463 (18) Å, respectively, from the least-squares plane of ring *C*. Bond lengths and angles in the whole mol­ecule are in characteristic ranges.

## 3. Supra­molecular features

**Figure 3:**
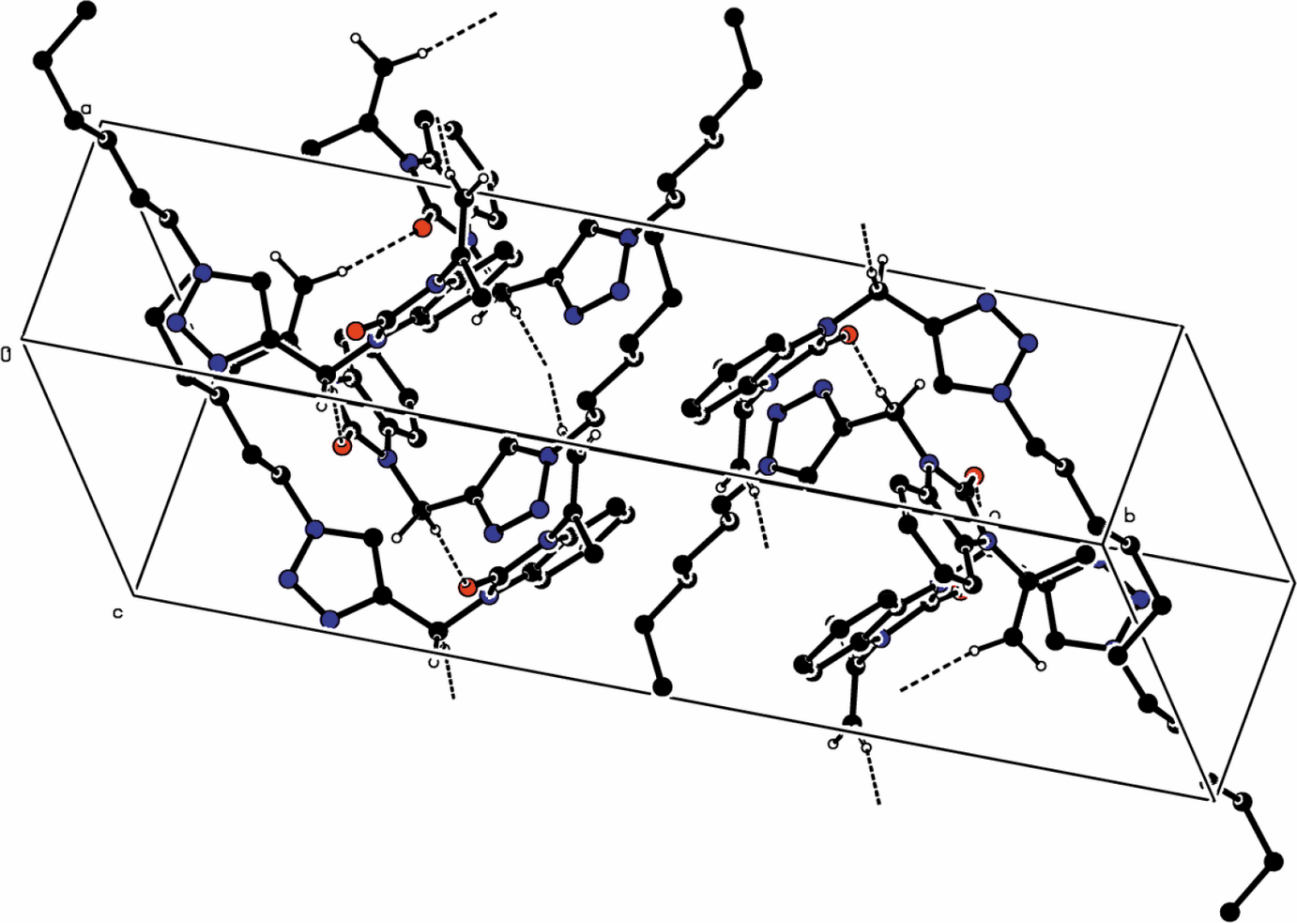
A partial packing diagram. Inter­molecular C—H⋯O hydrogen bonds are shown as dashed lines.

In the crystal, C—H⋯O hydrogen bonds link the mol­ecules into a network structure (Table 1 ▸, Fig. 3 ▸). There are no significant π–π inter­actions present, but two weak C—H⋯π(ring) inter­actions (Table 1 ▸) are observed.

## 4. Hirshfeld surface analysis

**Figure 4:**
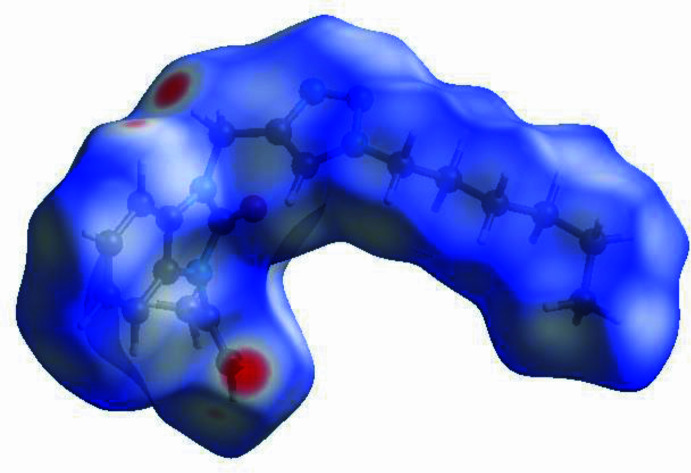
View of the three-dimensional Hirshfeld surface of the title compound plotted over *d*_norm_.

**Figure 5:**
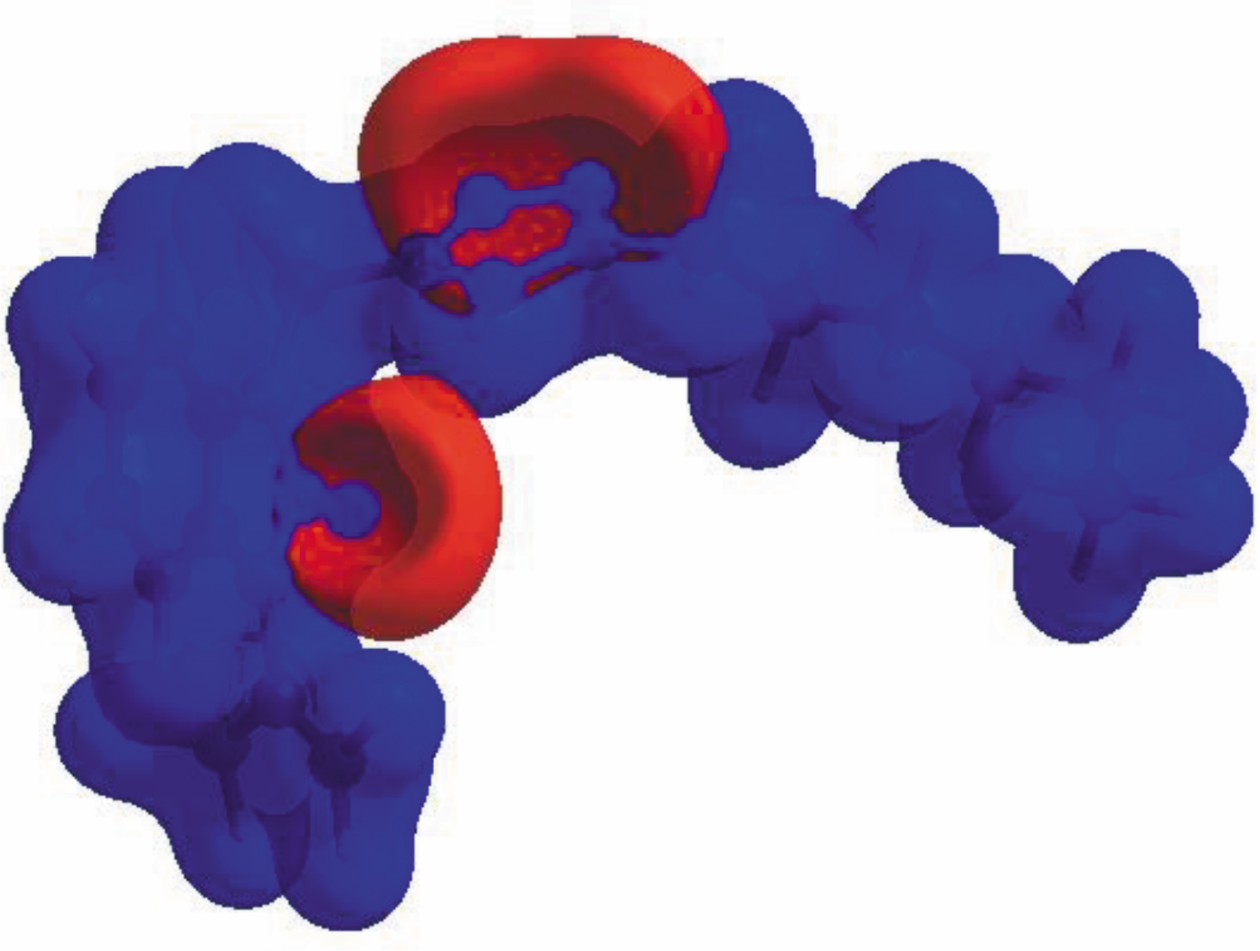
View of the three-dimensional Hirshfeld surface of the title compound plotted over electrostatic potential energy using the STO-3 G basis set at the Hartree–Fock level of theory. Hydrogen-bond donors and acceptors are shown as blue and red regions, respectively, around the atoms corresponding to positive and negative potentials.

**Figure 6:**
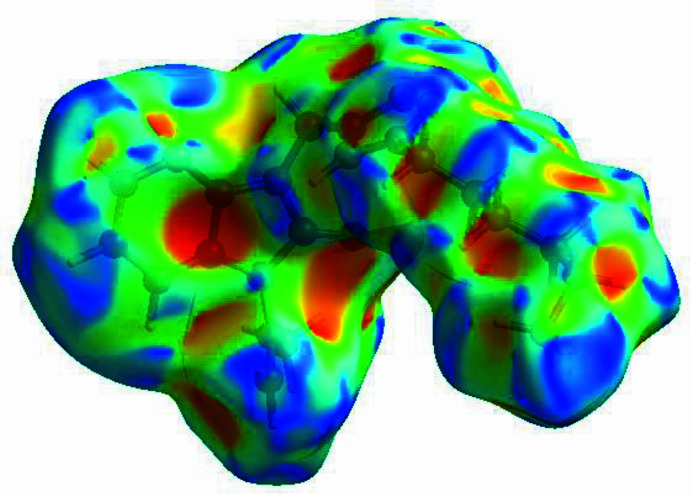
Hirshfeld surface of the title compound plotted over shape-index.

**Figure 7:**
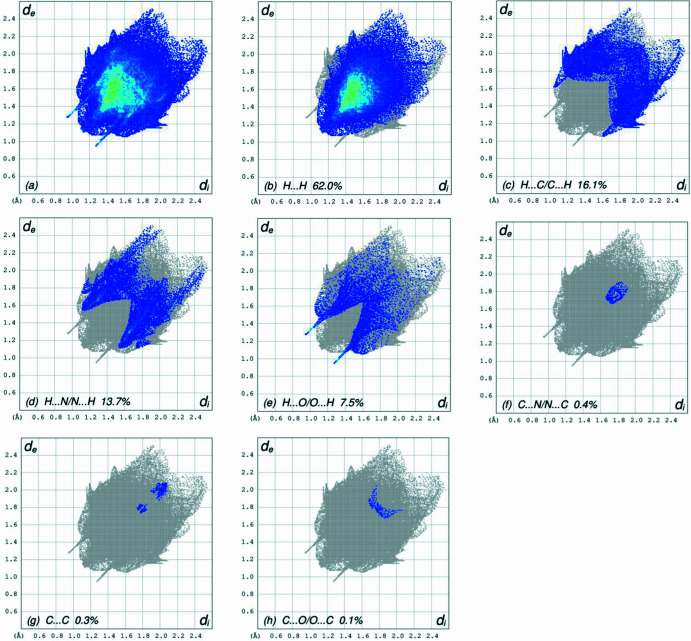
The full two-dimensional fingerprint plots for the title compound, showing (*a*) all inter­actions, and delineated into (*b*) H⋯H, (*c*) H⋯C/C⋯H, (*d*) H⋯N/N⋯H, (*e*) H⋯O/O⋯H, (*f*) C⋯N/N⋯C, (*g*) C⋯C and (*h*) C⋯O/O⋯C inter­actions. The *d*_i_ and *d*_e_ values are the closest inter­nal and external distances (in Å) from given points on the Hirshfeld surface.

In order to qu­antify the inter­molecular inter­actions in the crystal of the title compound, a Hirshfeld surface (HS) analysis (Hirshfeld, 1977 ▸; Spackman & Jayatilaka, 2009 ▸) was carried out using *CrystalExplorer* (Spackman *et al.*, 2021 ▸). It is noted that only the major occupancy component of the disordered atoms at the terminal propyl moiety of the hexyl chain were taken into account for the analysis. In the HS plotted over *d*_norm_ (Fig. 4 ▸), the white surface indicates contacts with distances equal to the sum of van der Waals radii, and the red and blue colours indicate distances shorter (in close contact) or longer (distant contact) than the van der Waals radii, respectively (Venkatesan *et al.*, 2016 ▸). The bright-red spots indicate their roles as the respective donors and/or acceptors; they also appear as blue and red regions corresponding to positive and negative potentials on the HS mapped over electrostatic potential (Spackman *et al.*, 2008 ▸; Jayatilaka *et al.*, 2005 ▸), as shown in Fig. 5 ▸. The blue regions indicate the positive electrostatic potential (hydrogen-bond donors), while the red regions indicate the negative electrostatic potential (hydrogen-bond acceptors). Possible π–π stacking and C—H⋯π inter­actions can also be visualized using the shape-index surface, which can be used to identify characteristic packing modes, in particular, planar stacking arrangements and the presence of aromatic stacking inter­actions. The shape-index surface represents the C—H⋯π inter­actions as red *p*-holes, which are related to the electron ring inter­actions between the CH groups with the centroid of the aromatic rings of neighbouring mol­ecules. Fig. 6 ▸ clearly shows that there are C—H⋯π inter­actions present in the crystal packing of the title compound. On the other hand, the shape-index of the HS is a tool to visualize π–π stacking by the presence of adjacent red and blue triangles. If there are no adjacent red and/or blue triangles, then there are no π–π inter­actions, as Fig. 6 ▸ clearly suggests. The overall two-dimensional fingerprint plot, Fig. 7 ▸*a*, and those delineated into H⋯H, H⋯C/C⋯H, H⋯N/N⋯H, H⋯O/O⋯H, C⋯N/N⋯C, C⋯C and C⋯O/O⋯C (McKinnon *et al.*, 2007 ▸) are illustrated in Fig. 7 ▸*b*–*h*, respectively, together with their relative contributions to the Hirshfeld surface. The most important inter­action is H⋯H (Table 2 ▸) contributing 62.0% to the overall crystal packing, which is reflected in Fig. 7 ▸*b* as widely scattered points of high density due to the large hydrogen content of the mol­ecule. As a result of the presence of C—H⋯π inter­actions (Table 1 ▸, Fig. 6 ▸), the H⋯C/C⋯H contacts (Table 2 ▸) contribute 16.1% to the overall crystal packing. H⋯N/N⋯H contacts (Fig. 7 ▸*d*) make a 13.7% contribution to the HS, and the H⋯O/O⋯H contacts (Table 3 ▸ and Fig. 7 ▸*e*) amount to 7.5% of the overall crystal packing. Finally, the C⋯N/N⋯C (Fig. 7 ▸*f*), C⋯C (Fig. 7 ▸*g*) and C⋯O/O⋯C (Fig. 7 ▸*h*) contacts with 0.4%, 0.3% and 0.1% contributions, respectively, to the HS play a minor role.

**Figure 8:**
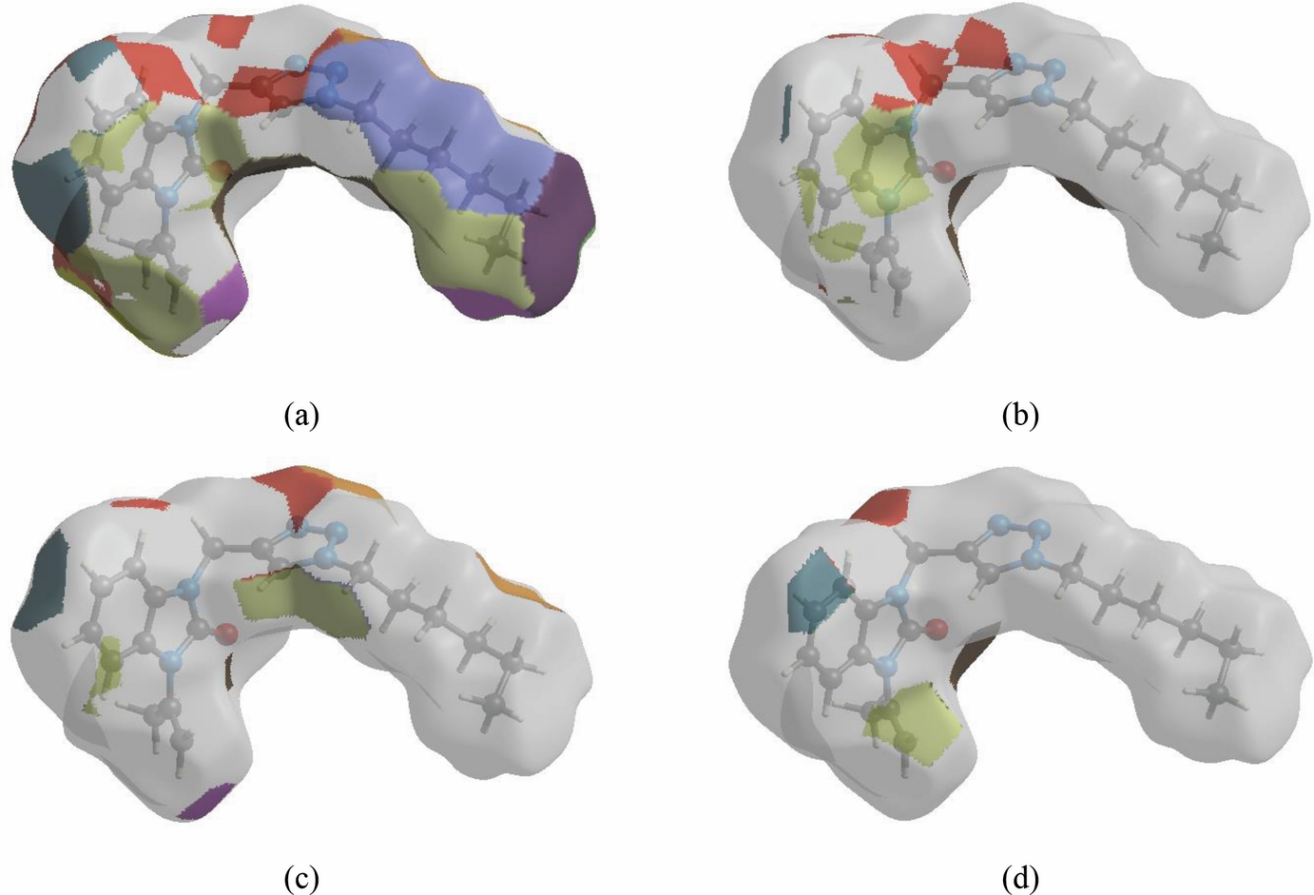
The Hirshfeld surface representation of contact patches plotted onto the surface for (*a*) H⋯H, (*b*) H⋯C/C⋯H, (*c*) H⋯N/N⋯H and H⋯O/O⋯H inter­actions.

The nearest neighbour environment of a mol­ecule can be determined from the colour patches on the HS based on how close to other mol­ecules they are. The Hirshfeld surface representations of contact patches plotted onto the surface are shown for the H⋯H, H⋯C/C⋯H, H⋯N/N⋯H and H⋯O/O⋯H inter­actions in Fig. 8 ▸*a*–*d*, respectively.

The Hirshfeld surface analysis confirms the importance of H-atom contacts in establishing the crystal packing, as shown by the large number of H⋯H, H⋯C/C⋯H, H⋯N/N⋯H and H⋯O/O⋯H inter­actions (Hathwar *et al.*, 2015 ▸).

## 5. Inter­action energy calculations and energy frameworks

**Figure 9:**
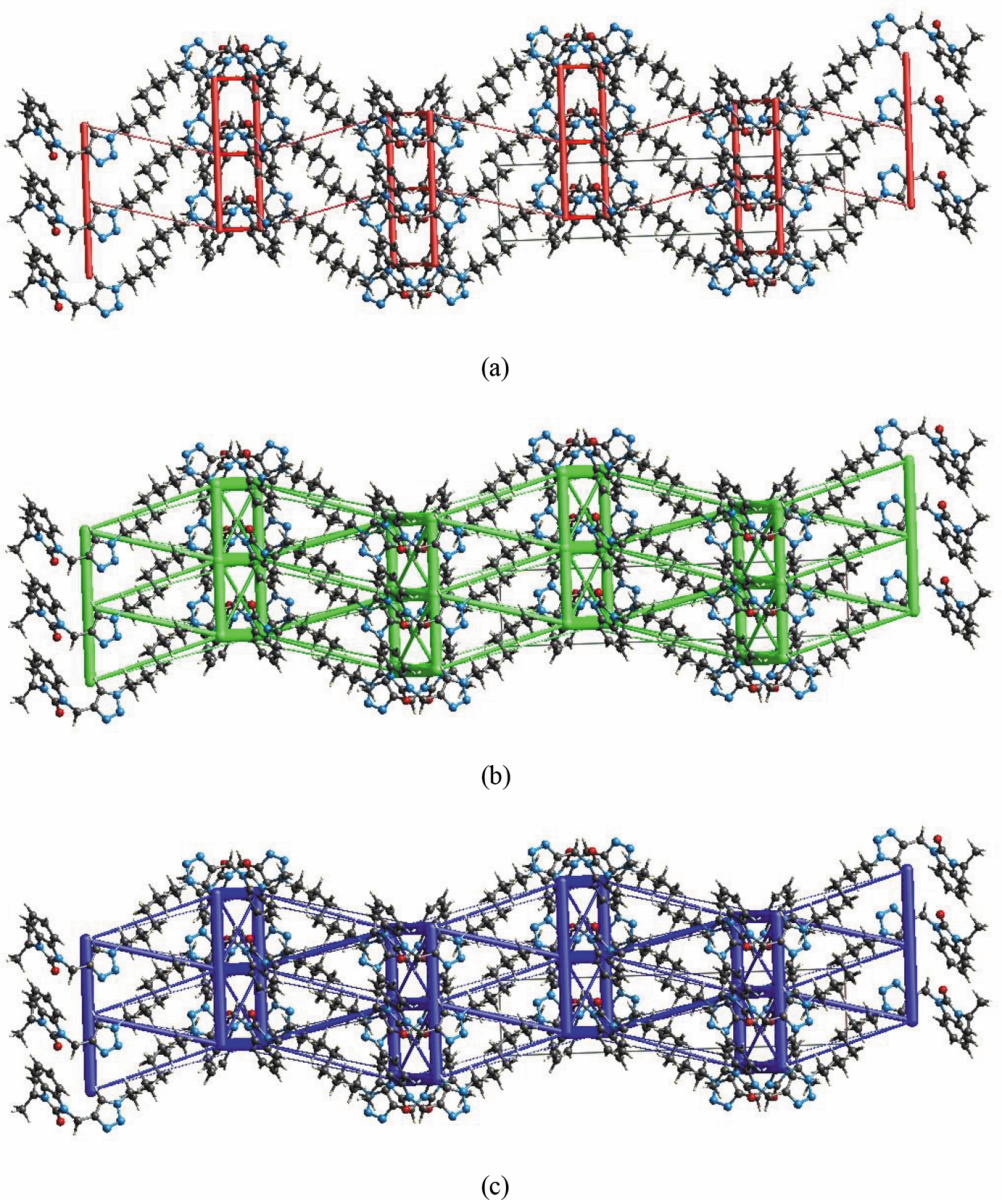
The energy frameworks for a cluster of mol­ecules of title compound viewed down the *c*-axis direction showing (*a*) electrostatic energy, (*b*) dispersion energy and (*c*) total energy diagrams. The cylindrical radius is proportional to the relative strength of the corresponding energies and they were adjusted to the same scale factor of 80 with cut-off value of 5 kJ mol^−1^ within 2×2×2 unit cells.

The inter­molecular inter­action energies were calculated using the CE–B3LYP/6–31G(d,p) energy model available in *CrystalExplorer* (Spackman *et al.*, 2021 ▸), where a cluster of mol­ecules is generated by applying crystallographic symmetry operations with respect to a selected central mol­ecule within the radius of 3.8 Å by default (Turner *et al.*, 2014 ▸). The total inter­molecular energy (*E*_tot_) is the sum of electrostatic (*E*_ele_), polarization (*E*_pol_), dispersion (*E*_dis_) and exchange-repulsion (*E*_rep_) energies (Turner *et al.*, 2015 ▸) with scale factors of 1.057, 0.740, 0.871 and 0.618, respectively (Mackenzie *et al.*, 2017 ▸). Hydrogen-bonding inter­action energies (in kJ mol^−1^) were calculated to be −32.5 (*E*_ele_), −9.2 (*E*_pol_), −60.5 (*E*_dis_), 54.9(*E*_rep_) and 59.9 (*E*_tot_) for the C10—H10*B*⋯O1, and −19.8 (*E*_ele_), −7.5 (*E*_pol_), −72.3 (*E*_dis_), 50.3 (*E*_rep_) and −58.3 (*E*_tot_) for the C11—H11*A*⋯O1 hydrogen-bonding inter­actions. Energy frameworks combine the calculation of inter­molecular inter­action energies with a graphical representation of their magnitude (Turner *et al.*, 2015 ▸). Energies between mol­ecular pairs are represented as cylinders joining the centroids of pairs of mol­ecules with the cylinder radius proportional to the relative strength of the corresponding inter­action energy. Energy frameworks were constructed for *E*_ele_ (red cylinders), *E*_dis_ (green cylinders) and *E*_tot_ (blue cylinders) (Fig. 9 ▸*a*–*c*). The evaluation of the electrostatic, dispersion and total energy frameworks indicate that the stabilization is dominated by the dispersion energy contributions in the crystal structure of the title compound.

## 6. Database survey

**Figure 10:**
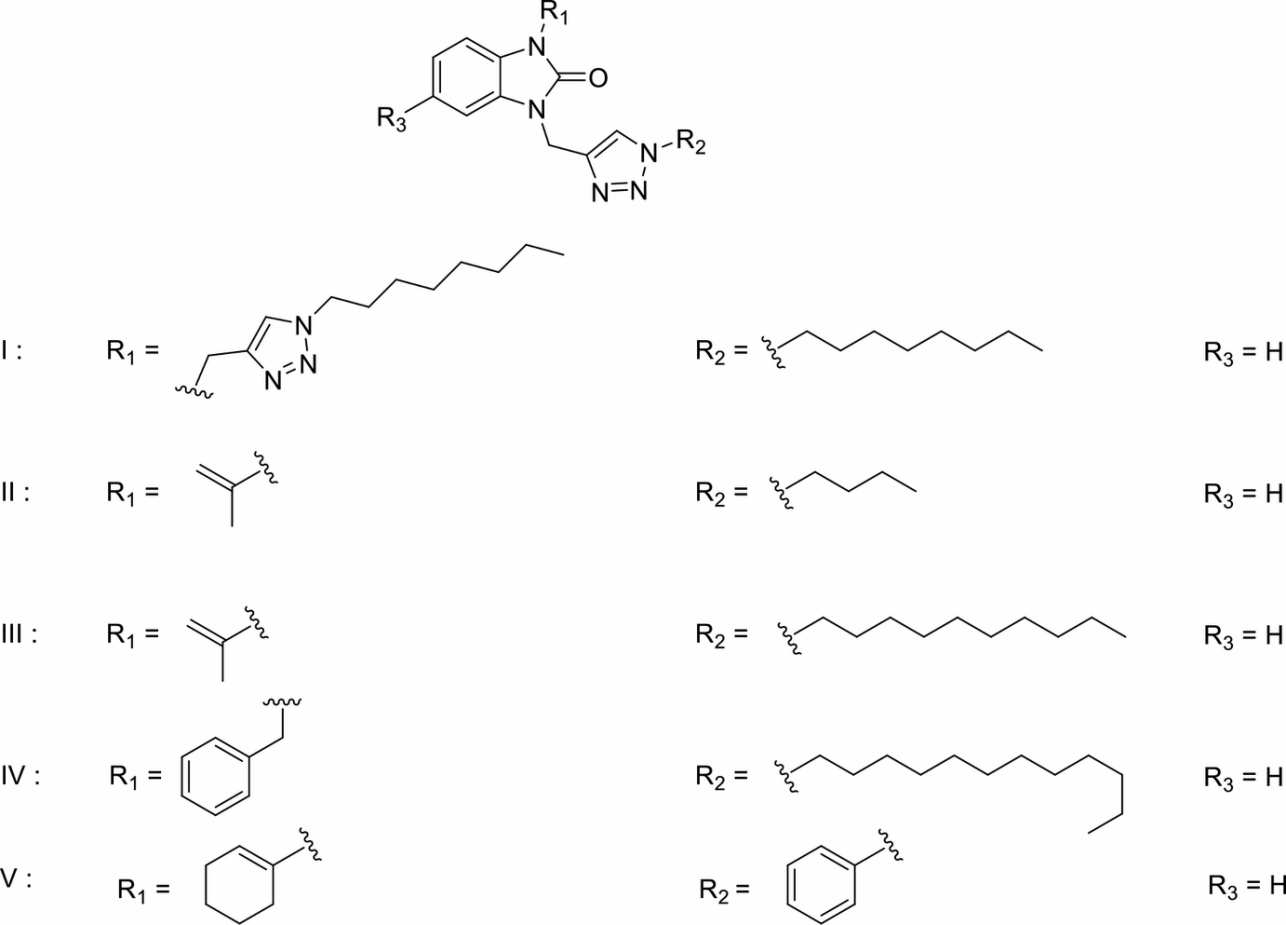
Related compounds.

A survey of the Cambridge Structural Database (CSD, updated July 2024; Groom *et al.*, 2016 ▸) found several mol­ecules that are similar to the title compound. These include: formula I in Fig. 10 ▸ (CSD refcode YIVWUZ; Zouhair *et al.*, 2023 ▸), formula II with *R*1 = –C(CH_3_)=CH_2_, *R*2 = –C_6_H_9_, and *R*3 = –H (CSD refcode ROPKOA; El Atrassi *et al.*, 2024 ▸), formula III with *R*_1_ = –C(CH_3_)=CH_2_, *R*_2_ = –C_10_H_22_, and *R*_3_ = –H (CSD refcode ETAJOB; Saber *et al.*, 2021 ▸), formula IV with *R*_1_ = –CH_2_C_6_H_5_, *R*_2_ = –C_12_H_26_, and *R*_3_ = –H (CSD refcode ETAKAO; Saber *et al.*, 2021 ▸) and formula V with *R*_1_ = –C_6_H_9_, *R*_2_ = –C_6_H_5_, and *R*_3_ = –H (CSD refcode PAZFOO; Adardour *et al.*, 2017 ▸). Most of the identified compounds exhibit an almost planar benzimidazol-2-one ring system, with the dihedral angle between the constituent rings being less than 1°, or the nitro­gen atom bearing the exocyclic substituent being less than 0.03 Å from the mean plane of the remaining nine atoms.

## 7. Synthesis and crystallization

2.87 mmol of compound **1** (Fig. 1 ▸) and 0.45 mmol of 1-azido­hexane were dissolved in 10 ml of ethanol. This solution was added into 1.64 mmol of CuSO_4_ and 3.73 mmol of sodium ascorbate, dissolved in 10 ml of distilled water. The reaction mixture was stirred for 10 h at room temperature. After filtration and concentration of the solution under reduced pressure, the obtained residue was chromatographed on a silica gel column using ethyl acetate/hexane (3/1 *v*/*v*) as the eluent. The resulting solid was filtered, washed with water, dried, and recrystallized from ethanol. The title compound **2** was obtained in a yield of 87%.

## 8. Refinement

Crystal data, data collection and structure refinement details are summarized in Table 3 ▸. The H10*A* and H10*B* hydrogen atoms were located in a difference-Fourier map, and were refined isotropically. The other C-bound hydrogen-atom positions were calculated geometrically at distances of 0.95 Å (for aromatic CH), 0.99 Å (for CH_2_) and 0.98 Å (for CH_3_) and refined using a riding model by applying the constraints *U*_iso_(H) = *k*×*U*_eq_(C), where *k* = 1.2 for CH and CH_2_ and *k* = 1.5 for CH_3_. The terminal propyl moiety of the hexyl chain is disordered over two positions (H17*A*, H17*B*, C18*A*, H18*A*, H18*B*, C19*A*, H19*A*, H19*B*, H19*C*, H17*C*, H17*D*, C18*B*, H18*C*, H18*D*, C19*B*, H19*D*, H19*E*, H19*F*) with a refined occupancy ratio of 0.821 (5):0.179 (5).

## Supplementary Material

Crystal structure: contains datablock(s) I. DOI: 10.1107/S2056989024008703/wm5732sup1.cif

Structure factors: contains datablock(s) I. DOI: 10.1107/S2056989024008703/wm5732Isup2.hkl

Supporting information file. DOI: 10.1107/S2056989024008703/wm5732Isup3.cdx

Supporting information file. DOI: 10.1107/S2056989024008703/wm5732Isup4.cml

CCDC reference: 2381865

Additional supporting information:  crystallographic information; 3D view; checkCIF report

## Figures and Tables

**Table 1:** Hydrogen-bond geometry (Å, °) *Cg*1 is the centroid of the C1–C6 ring.

*D*—H⋯*A*	*D*—H	H⋯*A*	*D*⋯*A*	*D*—H⋯*A*
C10—H10*B*⋯O1^iv^	0.99 (2)	2.31 (2)	3.284 (2)	168.8 (18)
C11—H11*A*⋯O1^ii^	0.99	2.37	3.3577 (17)	173
C11—H11*B*⋯*Cg*1^i^	0.99	2.76	3.5082 (18)	135
C15—H15*B*⋯*Cg*1^ii^	0.99	2.88	3.7599 (19)	152

Symmetry codes: (i) 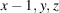; (ii) 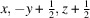; (iv) 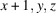.

**Table 2:** Selected interatomic distances (Å)

O1⋯C9	3.185 (2)	C7⋯H9*C*	2.85
O1⋯H10*B*^i^	2.31 (2)	H3⋯C7^ii^	2.80
O1⋯H11*B*	2.72	C10⋯H9*C*^iii^	2.80
O1⋯H9*C*	2.60	H16*B*⋯H19*A*	2.41
H3⋯O1^ii^	2.69	H17*A*⋯H19*A*^i^	2.36
H11*A*⋯O1^ii^	2.37	H17*B*⋯H19*C*	2.41
C3⋯H11*A*	2.87		

Symmetry codes: (i) 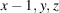; (ii) 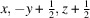; (iii) 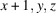.

**Table 3:** Experimental details

Crystal data	
Chemical formula	C_19_H_25_N_5_O
*M*_r_	339.44
Crystal system, space group	Monoclinic, *P*2_1_/*c*
Temperature (K)	160
*a*, *b*, *c* (Å)	5.7820 (3), 26.5057 (14), 11.7704 (5)
β (°)	90.407 (4)
*V* (Å^3^)	1803.84 (15)
*Z*	4
Radiation type	Cu *K*α
μ (mm^−1^)	0.64
Crystal size (mm)	0.25 × 0.14 × 0.09

Data collection	
Diffractometer	SuperNova, Dual, Cu at home/near, Atlas
Absorption correction	Analytical [*CrysAlis PRO* (Rigaku OD, 2023 ▸) based on expressions derived by Clark & Reid (1995 ▸)]
*T*_min_, *T*_max_	0.901, 0.951
No. of measured, independent and observed [*I* > 2σ(*I*)] reflections	21177, 3808, 3160
*R*_int_	0.052
(sin θ/λ)_max_ (Å^−1^)	0.632

Refinement	
*R*[*F*^2^ > 2σ(*F*^2^)], *wR*(*F*^2^), *S*	0.045, 0.127, 1.03
No. of reflections	3808
No. of parameters	257
No. of restraints	42
H-atom treatment	H atoms treated by a mixture of independent and constrained refinement
Δρ_max_, Δρ_min_ (e Å^−3^)	0.25, −0.26

Computer programs: *CrysAlis PRO* (Rigaku OD, 2023 ▸), *SHELXT* (Sheldrick, 2015*a* ▸), *SHELXL* (Sheldrick, 2015*b* ▸), *OLEX2* (Dolomanov *et al.*, 2009 ▸) and *publCIF* (Westrip, 2010 ▸).

## supplementary crystallographic information

### 1-[(1-Hexyl-1H-1,2,3-triazol-4-yl)methyl]-3-(1-methylethenyl)benzimidazol-2-one Crystal data

**Table:**

C_19_H_25_N_5_O	*F*(000) = 728
*M**_r_* = 339.44	*D*_x_ = 1.250 Mg m^−^^3^
Monoclinic, *P*2_1_/*c*	Cu *K*α radiation, λ = 1.54184 Å
*a* = 5.7820 (3) Å	Cell parameters from 8532 reflections
*b* = 26.5057 (14) Å	θ = 3.3–76.2°
*c* = 11.7704 (5) Å	µ = 0.64 mm^−^^1^
β = 90.407 (4)°	*T* = 160 K
*V* = 1803.84 (15) Å^3^	Plate, colourless
*Z* = 4	0.25 × 0.14 × 0.09 mm

### 1-[(1-Hexyl-1H-1,2,3-triazol-4-yl)methyl]-3-(1-methylethenyl)benzimidazol-2-one Data collection

**Table:**

SuperNova, Dual, Cu at home/near, Atlas diffractometer	3808 independent reflections
Radiation source: micro-focus sealed X-ray tube, SuperNova (Cu) X-ray Source	3160 reflections with *I* > 2σ(*I*)
Mirror monochromator	*R*_int_ = 0.052
Detector resolution: 10.3801 pixels mm^-1^	θ_max_ = 77.0°, θ_min_ = 3.3°
ω scans	*h* = −7→7
Absorption correction: analytical [CrysAlisPro (Rigaku OD, 2023) based on expressions derived by Clark & Reid (1995)]	*k* = −33→29
*T*_min_ = 0.901, *T*_max_ = 0.951	*l* = −14→10
21177 measured reflections	

### 1-[(1-Hexyl-1H-1,2,3-triazol-4-yl)methyl]-3-(1-methylethenyl)benzimidazol-2-one Refinement

**Table:**

Refinement on *F*^2^	Hydrogen site location: mixed
Least-squares matrix: full	H atoms treated by a mixture of independent and constrained refinement
*R*[*F*^2^ > 2σ(*F*^2^)] = 0.045	*w* = 1/[σ^2^(*F*_o_^2^) + (0.0636*P*)^2^ + 0.5249*P*] where *P* = (*F*_o_^2^ + 2*F*_c_^2^)/3
*wR*(*F*^2^) = 0.127	(Δ/σ)_max_ < 0.001
*S* = 1.03	Δρ_max_ = 0.25 e Å^−^^3^
3808 reflections	Δρ_min_ = −0.26 e Å^−^^3^
257 parameters	Extinction correction: SHELXL (Sheldrick, 2015b), Fc^*^=kFc[1+0.001xFc^2^λ^3^/sin(2θ)]^-1/4^
42 restraints	Extinction coefficient: 0.0036 (4)
Primary atom site location: dual	

### 1-[(1-Hexyl-1H-1,2,3-triazol-4-yl)methyl]-3-(1-methylethenyl)benzimidazol-2-one Special details

**Table:**

Geometry. All esds (except the esd in the dihedral angle between two l.s. planes) are estimated using the full covariance matrix. The cell esds are taken into account individually in the estimation of esds in distances, angles and torsion angles; correlations between esds in cell parameters are only used when they are defined by crystal symmetry. An approximate (isotropic) treatment of cell esds is used for estimating esds involving l.s. planes.
Refinement. 1. Fixed Uiso At 1.2 times of: All C(H) groups, All C(H,H) groups, All C(H,H,H,H) groups At 1.5 times of: All C(H,H,H) groups 2. Restrained distances C18B-C19B ~ C19A-C18A ~ C18B-C17 ~ C18A-C17 with sigma of 0.005 3. Uiso/Uaniso restraints and constraints C18A ~ C19A ~ C18B ~ C19B: within 3A with sigma of 0.01 and sigma for terminal atoms of 0.02 within 3A 4. Others Sof(H17C)=Sof(H17D)=Sof(C18B)=Sof(H18C)=Sof(H18D)=Sof(C19B)=Sof(H19D)= Sof(H19E)=Sof(H19F)=1-FVAR(1) Sof(H17A)=Sof(H17B)=Sof(C18A)=Sof(H18A)=Sof(H18B)=Sof(C19A)=Sof(H19A)= Sof(H19B)=Sof(H19C)=FVAR(1) 5.a Secondary CH2 refined with riding coordinates: C11(H11A,H11B), C14(H14A,H14B), C15(H15A,H15B), C16(H16A,H16B), C17(H17A, H17B), C17(H17C,H17D), C18A(H18A,H18B), C18B(H18C,H18D) 5.b Aromatic/amide H refined with riding coordinates: C3(H3), C4(H4), C5(H5), C6(H6), C13(H13) 5.c Idealised Me refined as rotating group: C9(H9A,H9B,H9C), C19A(H19A,H19B,H19C), C19B(H19D,H19E,H19F)

### 1-[(1-Hexyl-1H-1,2,3-triazol-4-yl)methyl]-3-(1-methylethenyl)benzimidazol-2-one Fractional atomic coordinates and isotropic or equivalent isotropic displacement parameters (Å^2^)

**Table:**

	*x*	*y*	*z*	*U*_iso_*/*U*_eq_	Occ. (<1)
C1	0.7940 (2)	0.17577 (5)	0.68604 (12)	0.0308 (3)	
O1	0.33103 (18)	0.21779 (4)	0.52367 (8)	0.0364 (3)	
N1	0.6614 (2)	0.17482 (5)	0.58607 (10)	0.0326 (3)	
N2	0.4944 (2)	0.22890 (5)	0.70261 (9)	0.0296 (3)	
C2	0.6863 (2)	0.20966 (5)	0.75975 (11)	0.0291 (3)	
N3	0.2200 (2)	0.35163 (5)	0.66222 (11)	0.0374 (3)	
C3	0.7721 (3)	0.21852 (6)	0.86806 (12)	0.0352 (3)	
H3	0.698298	0.241366	0.918318	0.042*	
N4	0.3129 (2)	0.39205 (5)	0.61517 (13)	0.0424 (3)	
C4	0.9712 (3)	0.19248 (7)	0.90008 (13)	0.0393 (4)	
H4	1.035084	0.197730	0.973712	0.047*	
C5	1.0783 (3)	0.15899 (6)	0.82652 (14)	0.0399 (4)	
H5	1.213610	0.141701	0.851127	0.048*	
N5	0.5415 (2)	0.38392 (5)	0.60927 (11)	0.0357 (3)	
C6	0.9922 (3)	0.15015 (6)	0.71767 (13)	0.0356 (3)	
H6	1.066630	0.127466	0.667251	0.043*	
C7	0.4775 (2)	0.20804 (6)	0.59617 (12)	0.0303 (3)	
C8	0.7004 (3)	0.14433 (6)	0.48692 (12)	0.0352 (3)	
C9	0.5113 (3)	0.10808 (7)	0.45822 (17)	0.0492 (4)	
H9A	0.493585	0.083842	0.520422	0.074*	
H9B	0.550058	0.090019	0.388245	0.074*	
H9C	0.366145	0.126581	0.447146	0.074*	
C10	0.8948 (3)	0.14965 (7)	0.43056 (15)	0.0442 (4)	
H10A	0.931 (4)	0.1277 (8)	0.3654 (18)	0.056 (6)*	
H10B	1.012 (4)	0.1741 (9)	0.4565 (18)	0.058 (6)*	
C11	0.3375 (3)	0.26838 (6)	0.74017 (12)	0.0323 (3)	
H11A	0.349085	0.271778	0.823779	0.039*	
H11B	0.176646	0.258618	0.720965	0.039*	
C12	0.3908 (2)	0.31811 (6)	0.68620 (11)	0.0301 (3)	
C13	0.5969 (3)	0.33859 (6)	0.65264 (13)	0.0340 (3)	
H13	0.746367	0.323922	0.658726	0.041*	
C14	0.6934 (3)	0.42141 (6)	0.55722 (15)	0.0424 (4)	
H14A	0.855524	0.409626	0.563088	0.051*	
H14B	0.681253	0.453526	0.599697	0.051*	
C15	0.6340 (3)	0.43082 (6)	0.43358 (15)	0.0421 (4)	
H15A	0.475025	0.444526	0.427921	0.051*	
H15B	0.637541	0.398416	0.391783	0.051*	
C16	0.8018 (3)	0.46769 (7)	0.37858 (17)	0.0480 (4)	
H16A	0.807003	0.499065	0.424150	0.058*	
H16B	0.958743	0.452764	0.379478	0.058*	
C17	0.7356 (4)	0.48077 (8)	0.25675 (17)	0.0586 (5)	
H17A	0.581533	0.497062	0.257699	0.070*	0.821 (5)
H17B	0.719383	0.448852	0.213761	0.070*	0.821 (5)
H17C	0.613356	0.506877	0.252606	0.070*	0.179 (5)
H17D	0.688090	0.450729	0.212404	0.070*	0.179 (5)
C18A	0.9007 (5)	0.51525 (10)	0.1912 (3)	0.0583 (8)	0.821 (5)
H18A	0.819476	0.529284	0.123958	0.070*	0.821 (5)
H18B	0.948434	0.543763	0.240390	0.070*	0.821 (5)
C19A	1.1136 (5)	0.48654 (11)	0.1530 (3)	0.0744 (10)	0.821 (5)
H19A	1.195578	0.473157	0.219640	0.112*	0.821 (5)
H19B	1.215944	0.509327	0.111307	0.112*	0.821 (5)
H19C	1.066583	0.458600	0.103448	0.112*	0.821 (5)
C18B	0.9733 (13)	0.5011 (6)	0.2206 (8)	0.058 (3)	0.179 (5)
H18C	1.005039	0.534040	0.257090	0.070*	0.179 (5)
H18D	1.097440	0.477164	0.242305	0.070*	0.179 (5)
C19B	0.960 (2)	0.5068 (5)	0.0915 (8)	0.061 (3)	0.179 (5)
H19D	0.966338	0.473423	0.055916	0.092*	0.179 (5)
H19E	1.090519	0.527234	0.065372	0.092*	0.179 (5)
H19F	0.814545	0.523467	0.070434	0.092*	0.179 (5)

### 1-[(1-Hexyl-1H-1,2,3-triazol-4-yl)methyl]-3-(1-methylethenyl)benzimidazol-2-one Atomic displacement parameters (Å^2^)

**Table:**

	*U*^11^	*U*^22^	*U*^33^	*U*^12^	*U*^13^	*U*^23^
C1	0.0308 (7)	0.0297 (7)	0.0318 (7)	−0.0014 (5)	0.0010 (5)	0.0029 (5)
O1	0.0368 (6)	0.0420 (6)	0.0302 (5)	0.0042 (5)	−0.0033 (4)	0.0014 (4)
N1	0.0334 (6)	0.0346 (7)	0.0297 (6)	0.0030 (5)	0.0000 (5)	−0.0028 (5)
N2	0.0316 (6)	0.0307 (6)	0.0266 (5)	0.0029 (5)	0.0013 (4)	0.0008 (5)
C2	0.0296 (7)	0.0292 (7)	0.0285 (6)	−0.0014 (5)	0.0013 (5)	0.0035 (5)
N3	0.0307 (6)	0.0369 (7)	0.0446 (7)	0.0047 (5)	0.0007 (5)	0.0037 (5)
C3	0.0370 (8)	0.0387 (8)	0.0300 (7)	−0.0022 (6)	0.0002 (6)	0.0017 (6)
N4	0.0321 (7)	0.0360 (7)	0.0590 (8)	0.0054 (5)	0.0015 (6)	0.0074 (6)
C4	0.0387 (8)	0.0443 (9)	0.0347 (7)	−0.0047 (7)	−0.0056 (6)	0.0082 (6)
C5	0.0324 (7)	0.0404 (9)	0.0468 (9)	0.0000 (6)	−0.0054 (6)	0.0115 (7)
N5	0.0300 (6)	0.0326 (7)	0.0445 (7)	0.0013 (5)	−0.0004 (5)	0.0008 (5)
C6	0.0330 (7)	0.0318 (8)	0.0420 (8)	0.0015 (6)	0.0005 (6)	0.0024 (6)
C7	0.0316 (7)	0.0314 (7)	0.0280 (6)	−0.0004 (6)	0.0021 (5)	0.0029 (5)
C8	0.0366 (8)	0.0344 (8)	0.0346 (7)	0.0044 (6)	−0.0017 (6)	−0.0050 (6)
C9	0.0455 (9)	0.0438 (10)	0.0582 (10)	−0.0026 (8)	−0.0012 (8)	−0.0141 (8)
C10	0.0405 (9)	0.0512 (10)	0.0411 (8)	0.0039 (7)	0.0032 (7)	−0.0106 (7)
C11	0.0324 (7)	0.0350 (8)	0.0296 (6)	0.0050 (6)	0.0052 (5)	0.0008 (6)
C12	0.0304 (7)	0.0326 (7)	0.0275 (6)	0.0039 (5)	−0.0004 (5)	−0.0026 (5)
C13	0.0290 (7)	0.0346 (8)	0.0385 (7)	0.0045 (6)	−0.0012 (6)	0.0013 (6)
C14	0.0358 (8)	0.0348 (8)	0.0567 (10)	−0.0045 (6)	−0.0001 (7)	0.0035 (7)
C15	0.0354 (8)	0.0372 (8)	0.0538 (9)	0.0006 (6)	0.0048 (7)	0.0046 (7)
C16	0.0436 (9)	0.0369 (9)	0.0636 (11)	−0.0008 (7)	0.0124 (8)	0.0025 (8)
C17	0.0648 (12)	0.0539 (12)	0.0574 (11)	−0.0017 (10)	0.0196 (9)	0.0042 (9)
C18A	0.0807 (19)	0.0386 (15)	0.0561 (17)	0.0058 (12)	0.0190 (14)	0.0133 (11)
C19A	0.0639 (17)	0.0589 (17)	0.101 (2)	−0.0032 (13)	0.0307 (16)	0.0218 (15)
C18B	0.093 (7)	0.039 (6)	0.042 (5)	−0.009 (5)	0.012 (5)	0.009 (4)
C19B	0.077 (7)	0.059 (6)	0.047 (5)	−0.002 (5)	0.011 (5)	0.012 (5)

### 1-[(1-Hexyl-1H-1,2,3-triazol-4-yl)methyl]-3-(1-methylethenyl)benzimidazol-2-one Geometric parameters (Å, º)

**Table:**

C1—N1	1.4000 (18)	C11—C12	1.496 (2)
C1—C2	1.398 (2)	C12—C13	1.370 (2)
C1—C6	1.381 (2)	C13—H13	0.9500
O1—C7	1.2257 (18)	C14—H14A	0.9900
N1—C7	1.3862 (18)	C14—H14B	0.9900
N1—C8	1.4386 (18)	C14—C15	1.514 (2)
N2—C2	1.3901 (18)	C15—H15A	0.9900
N2—C7	1.3723 (18)	C15—H15B	0.9900
N2—C11	1.4560 (18)	C15—C16	1.525 (2)
C2—C3	1.385 (2)	C16—H16A	0.9900
N3—N4	1.3219 (19)	C16—H16B	0.9900
N3—C12	1.3567 (18)	C16—C17	1.522 (3)
C3—H3	0.9500	C17—H17A	0.9900
C3—C4	1.392 (2)	C17—H17B	0.9900
N4—N5	1.3414 (18)	C17—H17C	0.9900
C4—H4	0.9500	C17—H17D	0.9900
C4—C5	1.389 (2)	C17—C18A	1.534 (3)
C5—H5	0.9500	C17—C18B	1.539 (5)
C5—C6	1.391 (2)	C18A—H18A	0.9900
N5—C13	1.343 (2)	C18A—H18B	0.9900
N5—C14	1.464 (2)	C18A—C19A	1.518 (3)
C6—H6	0.9500	C19A—H19A	0.9800
C8—C9	1.492 (2)	C19A—H19B	0.9800
C8—C10	1.317 (2)	C19A—H19C	0.9800
C9—H9A	0.9800	C18B—H18C	0.9900
C9—H9B	0.9800	C18B—H18D	0.9900
C9—H9C	0.9800	C18B—C19B	1.528 (5)
C10—H10A	0.99 (2)	C19B—H19D	0.9800
C10—H10B	0.99 (2)	C19B—H19E	0.9800
C11—H11A	0.9900	C19B—H19F	0.9800
C11—H11B	0.9900		
			
O1···C9	3.185 (2)	C7···H9C	2.85
O1···H10B^i^	2.31 (2)	H3···C7^ii^	2.80
O1···H11B	2.72	C10···H9C^iii^	2.80
O1···H9C	2.60	H16B···H19A	2.41
H3···O1^ii^	2.69	H17A···H19A^i^	2.36
H11A···O1^ii^	2.37	H17B···H19C	2.41
C3···H11A	2.87		
			
C2—C1—N1	106.82 (12)	C12—C13—H13	127.5
C6—C1—N1	131.78 (14)	N5—C14—H14A	109.1
C6—C1—C2	121.40 (14)	N5—C14—H14B	109.1
C1—N1—C8	127.15 (12)	N5—C14—C15	112.42 (14)
C7—N1—C1	109.41 (12)	H14A—C14—H14B	107.9
C7—N1—C8	123.43 (12)	C15—C14—H14A	109.1
C2—N2—C11	127.86 (12)	C15—C14—H14B	109.1
C7—N2—C2	110.20 (12)	C14—C15—H15A	109.2
C7—N2—C11	121.76 (12)	C14—C15—H15B	109.2
N2—C2—C1	107.03 (12)	C14—C15—C16	111.90 (15)
C3—C2—C1	121.46 (14)	H15A—C15—H15B	107.9
C3—C2—N2	131.51 (14)	C16—C15—H15A	109.2
N4—N3—C12	108.69 (12)	C16—C15—H15B	109.2
C2—C3—H3	121.5	C15—C16—H16A	109.0
C2—C3—C4	117.09 (14)	C15—C16—H16B	109.0
C4—C3—H3	121.5	H16A—C16—H16B	107.8
N3—N4—N5	107.16 (12)	C17—C16—C15	112.93 (16)
C3—C4—H4	119.3	C17—C16—H16A	109.0
C5—C4—C3	121.32 (14)	C17—C16—H16B	109.0
C5—C4—H4	119.3	C16—C17—H17A	108.0
C4—C5—H5	119.2	C16—C17—H17B	108.0
C4—C5—C6	121.57 (15)	C16—C17—H17C	112.4
C6—C5—H5	119.2	C16—C17—H17D	112.4
N4—N5—C13	110.89 (13)	C16—C17—C18A	117.2 (2)
N4—N5—C14	120.47 (13)	C16—C17—C18B	97.0 (4)
C13—N5—C14	128.61 (13)	H17A—C17—H17B	107.2
C1—C6—C5	117.16 (14)	H17C—C17—H17D	109.9
C1—C6—H6	121.4	C18A—C17—H17A	108.0
C5—C6—H6	121.4	C18A—C17—H17B	108.0
O1—C7—N1	126.94 (13)	C18B—C17—H17C	112.4
O1—C7—N2	126.51 (14)	C18B—C17—H17D	112.4
N2—C7—N1	106.53 (12)	C17—C18A—H18A	109.4
N1—C8—C9	115.25 (13)	C17—C18A—H18B	109.4
C10—C8—N1	119.20 (15)	H18A—C18A—H18B	108.0
C10—C8—C9	125.55 (15)	C19A—C18A—C17	111.0 (2)
C8—C9—H9A	109.5	C19A—C18A—H18A	109.4
C8—C9—H9B	109.5	C19A—C18A—H18B	109.4
C8—C9—H9C	109.5	C18A—C19A—H19A	109.5
H9A—C9—H9B	109.5	C18A—C19A—H19B	109.5
H9A—C9—H9C	109.5	C18A—C19A—H19C	109.5
H9B—C9—H9C	109.5	H19A—C19A—H19B	109.5
C8—C10—H10A	120.9 (13)	H19A—C19A—H19C	109.5
C8—C10—H10B	120.1 (13)	H19B—C19A—H19C	109.5
H10A—C10—H10B	118.8 (17)	C17—C18B—H18C	110.6
N2—C11—H11A	109.2	C17—C18B—H18D	110.6
N2—C11—H11B	109.2	H18C—C18B—H18D	108.7
N2—C11—C12	111.94 (11)	C19B—C18B—C17	105.7 (7)
H11A—C11—H11B	107.9	C19B—C18B—H18C	110.6
C12—C11—H11A	109.2	C19B—C18B—H18D	110.6
C12—C11—H11B	109.2	C18B—C19B—H19D	109.5
N3—C12—C11	120.90 (13)	C18B—C19B—H19E	109.5
N3—C12—C13	108.30 (13)	C18B—C19B—H19F	109.5
C13—C12—C11	130.80 (13)	H19D—C19B—H19E	109.5
N5—C13—C12	104.97 (13)	H19D—C19B—H19F	109.5
N5—C13—H13	127.5	H19E—C19B—H19F	109.5
			
C1—N1—C7—O1	177.74 (14)	C4—C5—C6—C1	−0.6 (2)
C1—N1—C7—N2	−0.87 (16)	N5—C14—C15—C16	−176.78 (13)
C1—N1—C8—C9	118.16 (17)	C6—C1—N1—C7	−179.65 (15)
C1—N1—C8—C10	−61.0 (2)	C6—C1—N1—C8	1.2 (3)
C1—C2—C3—C4	0.4 (2)	C6—C1—C2—N2	179.77 (13)
N1—C1—C2—N2	−0.88 (15)	C6—C1—C2—C3	−0.8 (2)
N1—C1—C2—C3	178.54 (13)	C7—N1—C8—C9	−60.8 (2)
N1—C1—C6—C5	−178.31 (15)	C7—N1—C8—C10	120.01 (18)
N2—C2—C3—C4	179.71 (15)	C7—N2—C2—C1	0.37 (16)
N2—C11—C12—N3	147.34 (13)	C7—N2—C2—C3	−178.97 (15)
N2—C11—C12—C13	−32.5 (2)	C7—N2—C11—C12	−74.12 (17)
C2—C1—N1—C7	1.10 (16)	C8—N1—C7—O1	−3.1 (2)
C2—C1—N1—C8	−178.02 (14)	C8—N1—C7—N2	178.29 (13)
C2—C1—C6—C5	0.9 (2)	C11—N2—C2—C1	−174.74 (13)
C2—N2—C7—O1	−178.31 (14)	C11—N2—C2—C3	5.9 (2)
C2—N2—C7—N1	0.30 (16)	C11—N2—C7—O1	−2.8 (2)
C2—N2—C11—C12	100.48 (16)	C11—N2—C7—N1	175.77 (12)
C2—C3—C4—C5	−0.2 (2)	C11—C12—C13—N5	179.73 (14)
N3—N4—N5—C13	−0.26 (18)	C12—N3—N4—N5	0.17 (17)
N3—N4—N5—C14	177.91 (14)	C13—N5—C14—C15	116.89 (18)
N3—C12—C13—N5	−0.12 (16)	C14—N5—C13—C12	−177.75 (15)
C3—C4—C5—C6	0.3 (2)	C14—C15—C16—C17	−175.78 (15)
N4—N3—C12—C11	−179.90 (13)	C15—C16—C17—C18A	−176.58 (17)
N4—N3—C12—C13	−0.03 (17)	C15—C16—C17—C18B	−161.5 (6)
N4—N5—C13—C12	0.23 (17)	C16—C17—C18A—C19A	76.2 (3)
N4—N5—C14—C15	−60.9 (2)	C16—C17—C18B—C19B	168.8 (9)

Symmetry codes: (i) *x*−1, *y*, *z*; (ii) *x*, −*y*+1/2, *z*+1/2; (iii) *x*+1, *y*, *z*.

### 1-[(1-Hexyl-1H-1,2,3-triazol-4-yl)methyl]-3-(1-methylethenyl)benzimidazol-2-one Hydrogen-bond geometry (Å, º)

*Cg*1 is the centroid of the C1–C6 ring.

**Table:**

*D*—H···*A*	*D*—H	H···*A*	*D*···*A*	*D*—H···*A*
C10—H10*B*···O1^iii^	0.99 (2)	2.31 (2)	3.284 (2)	168.8 (18)
C11—H11*A*···O1^ii^	0.99	2.37	3.3577 (17)	173
C11—H11*B*···*Cg*1^i^	0.99	2.76	3.5082 (18)	135
C15—H15*B*···*Cg*1^ii^	0.99	2.88	3.7599 (19)	152

Symmetry codes: (i) *x*−1, *y*, *z*; (ii) *x*, −*y*+1/2, *z*+1/2; (iii) *x*+1, *y*, *z*.
